# No evidence of vertical transmission of SARS-CoV-2 after induction of labour in an immune-suppressed SARS-CoV-2-positive patient

**DOI:** 10.1136/bcr-2020-235581

**Published:** 2020-06-30

**Authors:** Koen Grimminck, Lindy Anne Maria Santegoets, Frederike Charlotte Siemens, Pieter Leendert Alex Fraaij, Irwin Karl Marcel Reiss, Sam Schoenmakers

**Affiliations:** 1 Department of Obstetrics and Gynaecology, Reinier de Graaf Gasthuis, Delft, Zuid-Holland, The Netherlands; 2 Department of Paediatrics, Subdivision Infectious Diseases and Immunology, Erasmus Medical Center - Sophia, Rotterdam, The Netherlands; 3 Department of Viroscience, Erasmus Medical Center, Rotterdam, The Netherlands; 4 Division of Neonatology, Erasmus Medical Center, Rotterdam, The Netherlands; 5 Department of Obstetrics and Gynaecology, Erasmus Medical Center, Rotterdam, Zuid-Holland, The Netherlands

**Keywords:** materno-fetal medicine, pregnancy, pneumonia (infectious disease), infectious diseases, global health

## Abstract

We present a case of a 38+1 weeks pregnant patient (G1P0) with a proven COVID-19 infection, who was planned for induction of labour because of pre-existent hypertension, systemic lupus erythematosus, respiratory problem of coughing and mild dyspnoea without fever during the COVID-19 pandemic in March 2020. To estimate the risk of vertical transmission of Severe Acute Respiratory Syndrome CoronaVirus 2 (SARS-CoV-2) during labour and delivery, we collected oropharyngeal, vaginal, urinary, placental and neonatal PCRs for SARS-CoV-2 during the period of admission. All PCRs, except for the oropharyngeal, were negative and vertical transmission was not observed. Labour and delivery were uncomplicated and the patient and neonate were discharged the next day. We give a short overview of the known literature about SARS-CoV-2-related infection during pregnancy, delivery and outcome of the neonate.

## Background

The pandemic, COVID-19, caused by the novel coronavirus SARS-CoV-2, originating from the Wuhan region of China, has spread rapidly throughout the world, including the Netherlands.^1 2^ COVID-19 pneumonia is a highly infectious disease and the ongoing outbreak has been declared a global public health emergency.^1^ In the Netherlands, the first COVID-19 patient was confirmed on the 27 February 2020. By the end of March 2020 in the Netherlands, 10 866 infected persons have been identified, of whom 3483 have been admitted to a hospital, more than 800 are currently admitted to an Intensive Care Unit (ICU) and 771 people died after COVID-19 confirmed infection.^3^

Due to the adaptive physiological and anatomical changes combined with the immunosuppressive state of pregnancy, pregnant women, in general, are prone for airway pathogens and pneumonia.^4^

Little is known about COVID-19 and pregnancy. So far, worldwide results have shown that clinical characteristics as well as CT imaging results of COVID-19 infection in pregnant women seem to be similar to non-pregnant adults.^5 6^ With the recent outbreak of the Zika virus in mind, we should be aware that new viruses can cross the placenta and cause congenital disease.^7^ So far, there seems to be limited evidence for vertical transmission of SARS-CoV-2 in pregnant patients.^5 8^ Schwartz *et al* described no vertical transmission in 38 pregnant women with COVID-19 and their neonates using PCR analyses.^9^ Unlike previous Middle East Respiratory Syndrome (MERS) and SARS infections in pregnant women, limited maternal deaths have been ascribed to COVID-19.^9 10^

With this case report, we aim to contribute to the evidence of the absence of transplacental and intrauterine transmission of SARS-CoV-2. We hereby report the outcome, management and investigation into the vertical transmission of a COVID-19 infection in a pregnant woman with pre-existent hypertension and systemic lupus erythematosus (SLE).

## Case presentation

In March 2020, a 31-year-old patient, G1P0, amenorrhea of 38+1 weeks, was scheduled for induction of labour because of pre-existent hypertension combined with a stable SLE with normal kidney function. Tests for Sjogrens Syndrome antibodies (SSA and SSB) were negative. The patient used methyldopa, prednisolone and azathioprine as prescribed medication. To reduce the risk of pre-eclampsia, acetylsalicylic acid was prescribed according to local protocol until 36 weeks of pregnancy.^11^ Fetal biometry was within normal range throughout pregnancy (antenatal ultrasounds for fetal biometrical parameters were performed at 28, 30, 34 and 36 weeks of gestation) with a continuous estimated fetal weight around the 16th percentile.

Due to the development of the progressive problem of coughing, the patient contacted our outpatient clinic before the scheduled induction of labour. Her history mentioned the daily use of prednisolone for SLE, did not reveal recent fever or having visited a known high-risk COVID-19 region or came in contact with people with a confirmed SARS-CoV-2 infection. After consulting the microbiologist, a PCR for SARS-CoV-2 was performed following the national protocol by collecting an oropharyngeal sample. The following day the result of the test was positive. To prevent further potential maternal respiratory distress, we decided to proceed with the scheduled induction of labour.

After a multidisciplinary consultation, the patient was admitted into an isolated zone on the delivery ward, following national and local COVID-19 guidelines.

On admission, physical examination revealed a temperature of 37.2°C, heart rate of 82 beats/min, blood pressure of 141/88 mm Hg, transcutaneous saturation of 99% by a FiO_2_ 0.21, with a respiratory rate of 12 breaths/min. Lung auscultation revealed no abnormal breath sounds. Laboratory findings were normal with a C-reactive protein of 14 mg/L, leucocytes of 6.5×10^9^/L, haemoglobin of 119.2 g/L, thrombocytes of 192×10^9^/L, neutrophils of 5.63×10^9^/L, lymphocytes of 0.22×10^9^/L, monocytes of 0.59×10^9^/L, creatinine of 38 µmol/L, estimated Glomerular Filtration Rate (eFGR) of >90 mL/min, uric acid of 0.18 mmol/L, Alanine aminotransferase (ALAT) of 20 U/L and Lactate dehydrogenase (LDH) of 203 U/L. After vaginal examination, a Foley catheter with 50cc of sterile water was placed intracervical to induce labour after which the patient went into labour. The patient received epidural analgesia to prevent maternal exhaustion and to have epidural access for extra analgesia in case of an emergency situation. Hereafter, the membranes were artificially broken and clear amniotic fluid was drained. Augmentation of labour by the administration of oxytocin was performed following local protocol until sufficient contractions (3–4 per 10 min) were established.^12^ A corticosteroid stress dose scheme was started following local protocol (100 mg in 30 min continued by 8.3 mg/hour until 8 hours post partum) because of the long-term systemic use of prednisolone with possible suppression of the hypothalamic–pituitary–adrenal axis.^13^ Two hours after artificial rupture of membranes, she progressed to 8 cm of dilation with the fetal head presenting at fetal station −3. We observed normal fetal heart tracing with stable maternal haemodynamic and respiratory parameters. One hour later the patient progressed into the second stage of labour. After 20 min, she delivered a daughter with an Apgar score of 9/10 at 5 and 10 min, respectively, an arterial umbilical pH level of 7.19 and a birth weight of 2880 g (30th percentile). The third stage of labour proceeded without complications.

There was a normal neonatal transitional phase after delivery, with no abnormal findings at physical examination. Antenatal SSA and SSB antibodies were tested negative and no signs of congenital abnormalities were noted. The neonate shared the room with both parents. Parents were advised to minimise physical contact during the symptomatic period with coughing, dyspnoea and/or a temperature above 38°C. During any physical contact within this period, the mother was advised to wear a surgical mask and surgical gloves to prevent transmission of SARS-CoV-2. Our patient bottle fed the neonate.

To be able to assess different anatomical compartments capable of viral shedding and different moments of possible vertical transmission during pregnancy and delivery, PCRs for SARS-CoV-2 were sampled. Prior to and directly after artificial rupture of membranes, the fornix posterior of the vagina was sampled, while a sample of catheter urine was collected during delivery. Post partum, both the maternal and the fetal side of the placenta were sampled in addition to an oropharyngeal PCR sample of the neonate. We were unable to collect a breastmilk sample. For the results, see table 1.

**Table 1:** PCR sampling for COVID-19 during different stages of labour

Swab	Date	Time	COVID-19
Maternal oropharyngeal	19-03-2020	09:00, at the outpatient clinic	Positive
Vaginal swab before rupture of membranes	20-03-2020	18:00	Negative
Vaginal swab after rupture of membranes	20-03-2020	22:30	Negative
Maternal urinary swab of catheter urine	20-03-2020	23:00	Negative
Placental swab maternal side	20-03-2020	23:45	Negative
Placental swab fetal side	20-03-2020	23:45	Negative
Neonatal oropharyngeal swab	21-03-2020	00.30	Negative

Twelve hours after delivery the patient was discharged from the hospital in good health and advised to stay isolated at home while being symptomatic for at least 14 days or until 24 hours had passed without problems.

## Outcome and follow-up

By using PCR samples for SARS-CoV-2 at different times and from different anatomical regions during delivery, we demonstrate no evidence of vertical transmission of SARS-CoV-2 after vaginal birth in an immune-suppressed SARS-CoV-2 positive patient with pre-existing hypertension and SLE. All PCR samples which could indicate vertical transmission (vaginal, maternal urine, maternal and fetal side of the placenta, oropharynx of the neonate) were tested negative. One week after delivery the patient still reported coughing and episodes of mild dyspnoea. There were no signs of neonatal infection with SARS-CoV-2. Four-week post partum, all maternal COVID-19-related symptoms were gone and there were no signs of a neonatal COVID-19 infection.

## Discussion

We report a term, prelabour, immuno-suppressed pregnant women with pre-existent hypertension and SLE who tested positive for SARS-CoV-2 and gave birth to a healthy female neonate. There was no evidence of transplacental or intrapartum transmission of SARS-CoV-2 following induction of labour in combination with administration of high doses of corticosteroids. An uneventful induction of labour, delivery, postpartum and postnatal course was observed.

During the course of the COVID-19 infection, several decisions had to be made based on the, at that moment, sparse available literature about COVID-19 and delivery. First, we decided to continue with the scheduled induction of labour, since she was at term with only mild respiratory symptoms, but at risk for respiratory insufficiency because of her immunocompromised state and the concomitant SARS-CoV-2 infection. Because of a lack of evidence of contra-indication for corticosteroids in patients with COVID-19 without respiratory insufficiency balanced against the known risk of an adrenal crisis without supportive therapy, we decided to provide our patient with a stress scheme of corticosteroids during the induction of labour.^14^ Due to the immunosuppressive medication during pregnancy and delivery, we argued for a possible higher risk of vertical transmission. The transplacental viral transmission was suggested by Dong *et al* because of the detection of elevated neonatal IgM and IgG antibodies directly post partum.^15^ This suggests vertical transmission and fetal infection since IgM antibodies cannot be transferred over the placenta. However, the reliability of IgM assays has been questioned.^16^ Depending on the routes and mechanism of intrauterine infection, further risk assessment can be made. If the transplacental transmission can occur, follow-up of fetal development after infection should be recommended. Viral shedding in diverse anatomical compartments (vaginal discharge, faeces, urine, amniotic fluid and haematogenous/placenta) could lead to fetal or neonatal infection during pregnancy, labour or delivery with possible consequences for the mode of delivery. The limited evidence of vertical transmission is comforting, but it is important to determine if and how, at which moments and in which situations, vertical transmission can occur. This will allow adequate advice for patients at risk and shared decision-making concerning delivery mode, antenatal and postnatal follow-up.

Royal College of Obstetricians and Gynaecologists (RCOG) guidelines recommend not to separate mother and child, with a maternal confirmed COVID-19 infection but to make use of adequate personal protective measurements to prevent transmission.^17^ Mother and child were discharged the next day to minimise the risk of spreading SARS-CoV-2 by shortening the hospital time. We provided strict instructions about COVID-19-related symptoms and when to contact the hospital. To identify the routes of and risks for possible vertical transmission during pregnancy and delivery, we advise to collect PCR samples for SARS-CoV-2 of different anatomical regions and at different time points during pregnancy and delivery. Immunoglobulins against SARS-CoV-2 should be tested for in maternal and neonatal blood as well as in breast milk after maternal infection.

Importantly, even less is known about the consequences of infection with COVID-19 in the first and second trimester of pregnancy. Recommendations are made to follow-up pregnant patients with COVID-19 because of the possible risk of abortion, premature delivery and fetal growth restriction as seen with other viral infections in the first and second trimester.^18–20^

**Learning points**

- Our case shows an uncomplicated vaginal birth in a patient with pre-existent hypertension and systemic lupus erythematosus (SLE) using long-term corticosteroids without signs of vertical transmission of SARS-CoV-2.
- The physical stress of vaginal birth did not influence dyspnoea and/or saturation levels in an immune-suppressed patient with pre-existent hypertension and SLE.
- In the absence of an obstetric or maternal contraindication, vaginal birth in COVID-19 patient’s wild mild respiratory symptoms can and should be considered.
- With personal protection measurements in place, COVID-19-positive mother and child should not be separated during the postpartum period. These measures may be sufficient to avoid horizontal transmission of the newborn as seen in our case.
- To interpret the vertical transmission of SARS-CoV-2, PCR samples for and immunoglobulins against SARS-CoV-2 should be included in the medical workup of pregnant women suspected for or with a confirmed COVID-19 infection. The same workup should be done for the newborn within the first few hours after birth.
